# Prevalence of bacteriologically confirmed pulmonary tuberculosis in South Africa, 2017–19: a multistage, cluster-based, cross-sectional survey

**DOI:** 10.1016/S1473-3099(22)00149-9

**Published:** 2022-08

**Authors:** Sizulu Moyo, Farzana Ismail, Martie Van der Walt, Nazir Ismail, Nkateko Mkhondo, Sicelo Dlamini, Thuli Mthiyane, Jeremiah Chikovore, Olanrewaju Oladimeji, David Mametja, Phaleng Maribe, Ishen Seocharan, Phumlani Ximiya, Irwin Law, Marina Tadolini, Khangelani Zuma, Samuel Manda, Charalambos Sismanidis, Yogan Pillay, Lindiwe Mvusi

**Affiliations:** a Human Sciences Research Council, Cape Town, South Africa; b School of Public Health and Family Medicine, University of Cape Town, Cape Town, South Africa; c Centre for Tuberculosis, National Institute for Communicable Diseases, Johannesburg, South Africa; d Department of Medical Microbiology, University of Pretoria, Pretoria, South Africa; e South African Medical Research Council, Cape Town, South Africa; f Global Tuberculosis Programme, World Health Organization, Geneva, Switzerland; g Tuberculosis Programme, World Health Organization, Pretoria, South Africa; h National Department of Health, Johannesburg, South Africa; i Health Professions Council of South Africa, Pretoria, South Africa; j Infectious Diseases Unit, IRCCS Azienda Ospedaliero-Universitaria di Bologna, Bologna, Italy; k Department of Medical and Surgical Sciences, Alma Mater Studiorum University of Bologna, Bologna, Italy; l Clinton Health Access Initiative, Pretoria, South Africa

## Abstract

**Background:**

Tuberculosis remains an important clinical and public health issue in South Africa, which has one of the highest tuberculosis burdens in the world. We aimed to estimate the burden of bacteriologically confirmed pulmonary tuberculosis among people aged 15 years or older in South Africa.

**Methods:**

This multistage, cluster-based, cross-sectional survey included eligible residents (age ≥15 years, who had slept in a house for ≥10 nights in the preceding 2 weeks) in 110 clusters nationally (cluster size of 500 people; selected by probability proportional-to-population size sampling). Participants completed face-to-face symptom questionnaires (for cough, weight loss, fever, and night sweats) and manually read digital chest X-ray screening. Screening was recorded as positive if participants had at least one symptom or an abnormal chest X-ray suggestive of tuberculosis, or a combination thereof. Sputum samples from participants who were screen-positive were tested by the Xpert MTB/RIF Ultra assay (first sample) and Mycobacteria Growth Indicator Tube culture (second sample), with optional HIV testing. Participants with a positive *Mycobacterium tuberculosis* complex culture were considered positive for bacteriologically confirmed pulmonary tuberculosis; when culture was not positive, participants with a positive Xpert MTB/RIF Ultra result with an abnormal chest X-ray suggestive of active tuberculosis and without current or previous tuberculosis were considered positive for bacteriologically confirmed pulmonary tuberculosis.

**Findings:**

Between Aug 15, 2017, and July 28, 2019, 68 771 people were enumerated from 110 clusters, with 53 250 eligible to participate in the survey, of whom 35 191 (66·1%) participated. 9066 (25·8%) of 35 191 participants were screen-positive and 234 (0·7%) were identified as having bacteriologically confirmed pulmonary tuberculosis. Overall, the estimated prevalence of bacteriologically confirmed pulmonary tuberculosis was 852 cases (95% CI 679–1026) per 100 000 population; the prevalence was highest in people aged 35–44 years (1107 cases [95% CI 703–1511] per 100 000 population) and those aged 65 years or older (1104 cases [680–1528] per 100 000 population). The estimated prevalence was approximately 1·6 times higher in men than in women (1094 cases [95% CI 835–1352] per 100 000 population *vs* 675 cases [494–855] per 100 000 population). 135 (57·7%) of 234 participants with tuberculosis screened positive by chest X-ray only, 16 (6·8%) by symptoms only, and 82 (35·9%) by both. 55 (28·8%) of 191 participants with tuberculosis with known HIV status were HIV-positive.

**Interpretation:**

Pulmonary tuberculosis prevalence in this survey was high, especially in men. Despite the ongoing burden of HIV, many participants with tuberculosis in this survey did not have HIV. As more than half of the participants with tuberculosis had an abnormal chest X-ray without symptoms, prioritising chest X-ray screening could substantially increase case finding.

**Funding:**

Global Fund, Bill & Melinda Gates Foundation, USAID.

## Introduction

The South African national tuberculosis control programme has long been active in responding to the country's tuberculosis burden. The country's response is guided by the national strategic plan's objectives and targets against HIV, tuberculosis, and sexually transmitted infections.^1^ South Africa was among the first countries to adopt and implement rapid molecular diagnosis with Xpert MTB/RIF assay technology.^2^ Furthermore, there are activities in the country to increase awareness about tuberculosis transmission and symptoms to drive screening and testing and improve case finding through various programmes, such as the Cheka Impilo and Welcome Back campaigns.^3^, ^4^ There is now a clear, consistent, and sustained downward trend in tuberculosis case notifications in South Africa, which is partly explained by high antiretroviral therapy (ART) coverage, with 62·3% of people living with HIV receiving ART in 2017.^5^, ^6^ However, the tuberculosis burden remains high, with an incidence estimated by WHO of 322 cases (95% CI 230–428) per 100 000 population in 2017, which placed South Africa as having one of the highest tuberculosis and tuberculosis and HIV co-infection burdens in the world.^6^

**Research in context**

**Evidence before this study**

South Africa is one of the 30 countries with high tuberculosis burden that, in 2020, collectively contributed to 86% of the estimated incident cases worldwide. The 2020 global tuberculosis report showed a large difference in the modelled estimates of the disease burden reported by WHO compared with the number of notified tuberculosis cases started on treatment. We searched PubMed for original research articles on national tuberculosis prevalence surveys in South Africa published in English between Jan 1, 2000, and Dec 31, 2020, using the terms ((“South Africa” AND (“2000/01/01”[PDat] : “2020/12/31”[PDat]))) AND (“tuberculosis prevalence” AND (“2000/01/01”[PDat] : “2020/12/31”[PDat])). We found no national-level population-based studies.

**Added value of this study**

This study refined the national estimate of the burden of pulmonary tuberculosis in South Africa and identified population groups in whom tuberculosis was underdiagnosed or under-reported. The survey found a high burden of tuberculosis, with a bimodal peak driven by HIV and recurrence of tuberculosis. Key diagnostic and reporting gaps were identified in young people (age 15–24 years), men, and older adults (age 65 years or older). Chest X-ray was identified as a key screening tool for increasing tuberculosis detection because many people with tuberculosis identified by chest X-ray did not report symptoms. In addition, as this was one of the first countries to use both Mycobacteria Growth Indicator Tube culture and Xpert MTB/RIF Ultra in a national tuberculosis prevalence survey, this study provided new insights into the use of Xpert MTB/RIF Ultra, resulting in a more nuanced case definition.

**Implications of all the available evidence**

The South African national tuberculosis control programme can develop more targeted interventions to address key gaps for greater impact in addressing the tuberculosis burden and consider adapting the current screening algorithm to include chest X-rays, to identify tuberculosis in those who are asymptomatic (ie, subclinical tuberculosis) or do not report typical symptoms. Future research should examine the effect of *Mycobacterium tuberculosis* infection in people who do not report symptoms on the overall burden of tuberculosis. Although Xpert MTB/RIF Ultra is a highly useful tool in tuberculosis prevalence surveys, it should be used in conjunction with culture because of the high false-positivity and low positive predictive value in active case finding (in screening populations with low prevalence of disease compared with those who present to health-care facilities). Therefore, an appropriate case definition considering all these factors is fundamental, with consideration of the different context from clinical settings.

We aimed to estimate the prevalence of bacteriologically confirmed pulmonary tuberculosis among people aged 15 years or older in South Africa and improve the understanding of tuberculosis epidemiology for evidence-based control efforts.

## Methods

### Study design and participants

**Figure 1:**
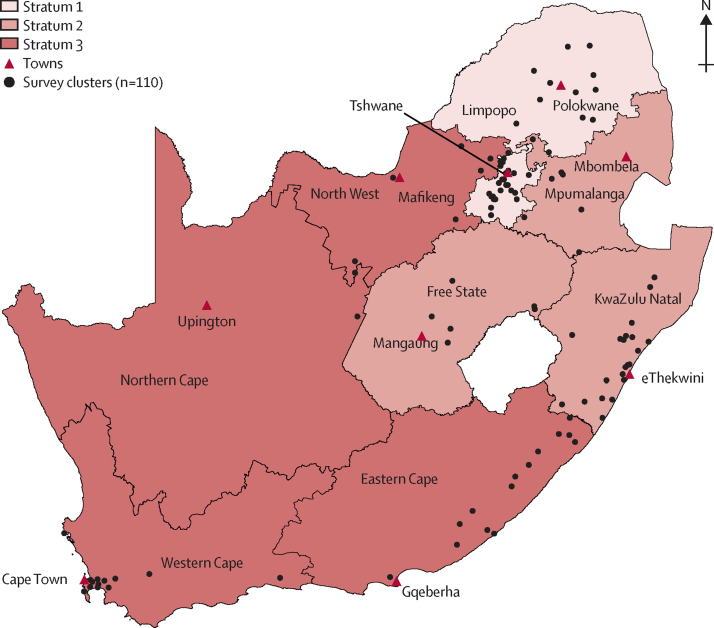
South African national tuberculosis prevalence survey map Black lines delineate provinces. The three strata are based on 2013 notification data. Stratum 1 (low tuberculosis prevalence) accounted for 38 clusters (Gauteng and Limpopo), stratum 2 (medium prevalence) had 29 clusters (KwaZulu-Natal, Mpumalanga, and Free State), and stratum 3 (high prevalence) had 44 clusters (Northern Cape, Western Cape, North West, and Eastern Cape). Data source: Human Sciences Research Council, 2020.

We did a multistage, cluster-based, cross-sectional survey designed according to WHO standards.^7^ We included 110 clusters, which were proportionally divided by population size across three strata in South Africa's nine provinces based on tuberculosis prevalence (low, medium, high) from 2013 notification data.^8^ Within each stratum, clusters were selected by means of multistage probability proportional-to-population size sampling, which was applied at provincial, district, and then subdistrict levels (figure 1). In each cluster, people aged 15 years or older who had slept in a house for at least 10 nights in the preceding 2 weeks were eligible to participate. No areas of the country were excluded from the sampling frame.

The survey protocol was approved by the South African Medical Research Council Research Ethics Committee in January, 2017 (EC001 2/2012), with annual renewal until completion. Individual written informed consent or assent and parent or guardian consent for participants younger than 18 years was obtained at survey enrolment. As tuberculosis is a notifiable disease in South Africa, participants also consented to give identifiers (names, address, telephone number) to facilitate follow-up of sputum sample results. These details were kept confidential by survey staff. Participation was voluntary, and participants received in-kind reimbursement to the value of US$5 (50 South African Rand) for time spent on survey activities.

Medical officers made medical referrals to the nearest health facility whenever indicated on the basis of the clinical picture or chest X-ray findings. Bacteriologically positive results (and identifiers) were sent to the national tuberculosis control programme through the tuberculosis coordinator of each cluster for follow-up and treatment initiation.

### Procedures

People in each cluster were enumerated by the field team to update the local population data. Thereafter, household heads completed a household questionnaire collecting sociodemographic details (appendix pp 5–17) and then received unique barcoded invitations for screening at a cluster screening site for eligible household members.

At the screening site, demographic details of eligible household members were verified using the barcoded invitations, and face-to-face individual questionnaires were completed. These included a four-symptom screen for cough (persistent of any duration), drenching night sweats, unexplained weight loss, and unexplained fever for at least 2 weeks (consistent with the 2014 South African tuberculosis management guidance^9^). Participants then had digital chest X-ray screening, unless they declined or a chest X-ray could not be done because of disability or pregnancy. Chest X-rays were read on site by a medical officer and were classified as normal, abnormal suggestive of tuberculosis, or abnormal not suggestive of tuberculosis. Participants with any screening symptoms or a chest X-ray classified as abnormal suggestive of tuberculosis, or a combination thereof, and those who did not report symptoms and had no chest X-ray, were asked to provide two sputum samples for laboratory testing. The first sample was taken immediately and the second was taken 1 h later. Participants who provided sputum samples were offered an optional HIV test (dried blood spot) in addition to optional self-reported HIV status collected during the interview. Those who accepted HIV testing received a barcoded voucher to retrieve the HIV test result at a designated clinic in the cluster with the necessary pretest and post-test counselling.

Sputum and dried blood spot samples were transported by courier daily under cold-chain conditions to the National Institute for Communicable Diseases Centre for Tuberculosis laboratory, Johannesburg, South Africa. The first sputum sample was tested with Xpert MTB/RIF Ultra assay (Cepheid, Sunnyvale, CA, USA) and the second was tested with liquid culture (Bactec MGIT 960, Becton Dickinson, Franklin Lakes, NJ, USA), considered by WHO to be the reference standard for tuberculosis diagnostics.^7^ Specimen processing followed the manufacturers’ instructions and standard operating procedures. For each batch of samples processed for culture, an H37Rv strain was included as a positive control and a non-inoculated Mycobacterial Growth Indicator Tube (MGIT) was used as a negative control. The culture positivity rate of smear-positive samples was 95%, as determined by routine samples processed by the laboratory, and the survey samples’ contamination rate was 5·4%; both indicators were within acceptable limits.^7^ Xpert MTB/RIF Ultra results were recorded as positive, negative, trace, or invalid. If the test was unsuccessful, the specimen was retested for a final result. For this survey, an Xpert MTB/RIF Ultra trace result was considered negative for *Mycobacterium tuberculosis* complex. At the time of survey design, there were minimal data regarding trace results, except for Dorman and colleagues’^10^ publication, which showed reduced specificity of Xpert MTB/RIF Ultra among those with trace results. In addition, trace results were not being reported as positive for *M tuberculosis* in the national tuberculosis programme, with which we aligned the survey results. Culture results were reported as positive, negative, or contaminated.

Dried blood spot samples were tested for HIV using a multiassay algorithm. Genscreen Ultra HIV Ag/Ab (BioRad, Hercules, CA, USA) was used as the screening assay. Specimens that showed a negative result were reported as negative, whereas those that showed a positive result were confirmed using the Murex HIV Ag/Ab Combination assay (Diasorin, Saluggia, Italy). Genscreen HIV-1 Western Blot assay (BioRad) was used in cases of discrepant results.

All chest X-rays classified by medical officers as abnormal suggestive of tuberculosis or abnormal not suggestive of tuberculosis, and 20% of those classified as normal, were read by an offsite radiologist as soon as possible. Discrepant readings were communicated to the medical officers, and sputum samples were collected where indicated if the survey team were still in that cluster and could easily access that participant again; otherwise the feedback guided future readings.

Screening was positive in the presence of at least one symptom or an abnormal chest X-ray, or a combination thereof. Participants who were screen-positive were grouped as positive by symptoms only, abnormal chest X-ray only, symptoms and abnormal chest X-ray, and no symptoms and without chest X-ray (for participants who reported no symptoms and declined or could not have a chest X-ray). Participants who were screen-positive and had *M tuberculosis* complex culture-positive sputum samples were considered positive for bacteriologically confirmed pulmonary tuberculosis; when culture was not positive (ie, negative, contaminated, or not done), screen-positive participants with Xpert MTB/RIF Ultra-positive samples and an abnormal chest X-ray (as determined by a central panel of three readers) and no history of current or previous tuberculosis, in keeping with emerging data at the time of the survey,^10^ were also considered as positive for bacteriologically confirmed pulmonary tuberculosis. HIV status was based on the dried blood spot result or on self-reported status.

Data were collected electronically using the REDCap system on tablet computers.^11^, ^12^ Centralised chest X-ray and laboratory data were captured directly onto REDCap. All data were backed up and linked into the central survey database. Data were cleaned throughout implementation, with final cleaning occurring before database lock.

### Statistical analysis

The sample size of 55 000 people with a cluster size of 500 was based on the assumptions of smear-positive tuberculosis prevalence estimated at 300 cases per 100 000 population (prevalence was assumed to be less than notification at the time of study design, given that disease duration is short in a population with high HIV prevalence), relative precision of 20%, design effect of 1·44, and a participation rate of 85%.

Data are summarised by frequencies, percentages, and medians as appropriate. We used WHO-recommended best-practice analytical methods to estimate the prevalence of bacteriologically confirmed pulmonary tuberculosis, which accounted for cluster sampling, non-participation, and missing data.^7^, ^13^ Specifically, cluster-level analysis and three individual-level logistic regression models were used. The model that was restricted to participants with multiple missing value imputation for individuals with missing outcome (the outcome variable that was imputed was tuberculosis status; ie, survey case, yes or no) and inverse probability weighting to represent all eligible individuals provided the single best estimate of tuberculosis prevalence at the population level. To avoid collinearity, and based on the number of observed survey cases, a finite number of the most important statistically independent variables was identified. The final imputation model to generate 25 datasets was defined using the following variables: stratum, age group, cough for longer than 2 weeks, past history of tuberculosis, HIV status, race, and sex (appendix pp 1–2). Survey prevalence was extrapolated to estimate prevalence for all forms of tuberculosis and for all ages in South Africa using WHO standard methodologies,^7^ and extrapolation was based on the proportion of the population that was younger than 15 years (29%), as per 2018 UN population estimates,^14^ the rate ratio of child to adult tuberculosis (0·6), and the proportion of notified tuberculosis cases that were extrapulmonary tuberculosis (9·7%), as reported by the national tuberculosis control programme for 2018.^6^ As an approximate indicator of case detection,^15^ the prevalence to case notification ratio was calculated by comparing prevalence rates with tuberculosis case notification rates of new and relapsed tuberculosis cases for the corresponding age groups and sex as reported by the national tuberculosis control programme for 2018. Data were analysed with STATA (version 15).

### Role of the funding source

The funders of the study had no role in study design, data collection, data analysis, data interpretation, or writing of the report.

## Results

**Figure 2:**
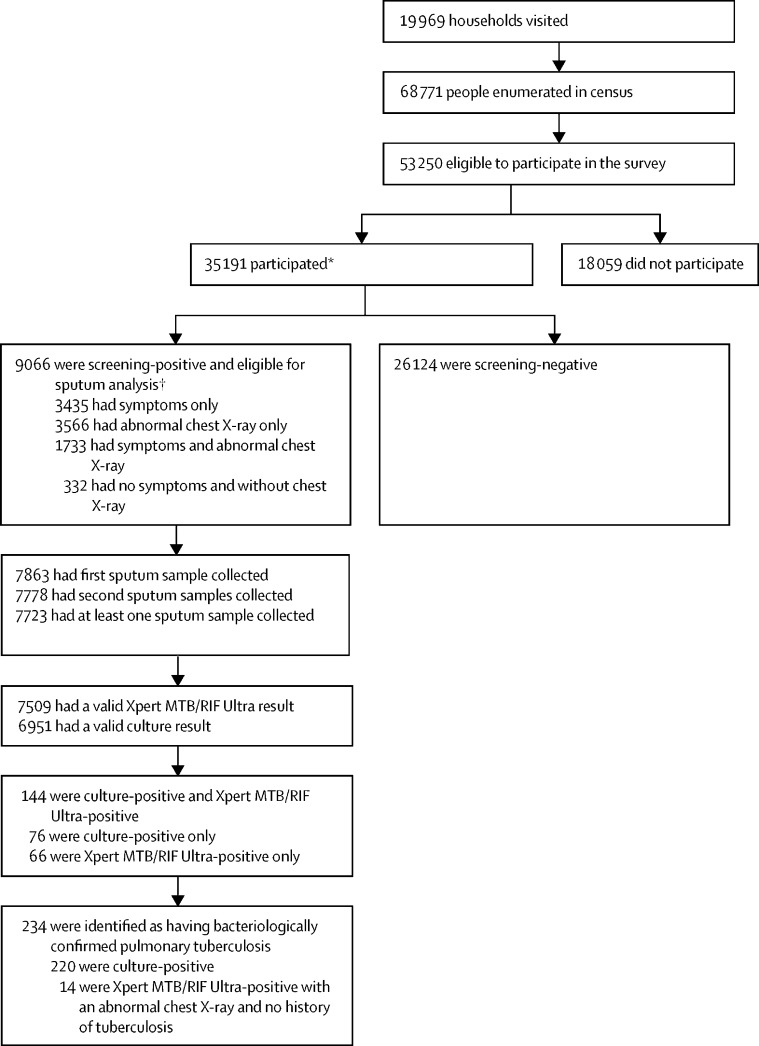
Study profile *26 350 provided self-reported HIV status (21 895 were negative and 4455 were positive). † 2176 had dried blood spot samples submitted for HIV testing (1851 were negative and 325 were positive).

Between Aug 15, 2017, and July 28, 2019, 68 771 people were enumerated from 19 969 households in 110 clusters, with 53 250 eligible to participate in the survey, of whom 35 191 (66·1%) participated (figure 2, table 1). The participation rate was higher in women than in men (21 803 [71·0%] of 30 689 women *vs* 13 388 [59·1%] of 22 561 men; p<0·0001). Participation rate varied significantly by age group and was highest in people aged 65 years or older (4449 [81·3%] of 5473) and lowest in those aged 25–34 years (7525 [59·6%] of 12 636; p<0·0001). Participation rate was higher in rural areas than in urban areas (15 708 [72·2%] of 21 769 people *vs* 19 483 [61·9%] of 31 481; p<0·0001).

**Table 1:** Survey participation by sex, age group, area of residence, and stratum

		**Eligible individuals**	**Participants**	**Participation rate**
Total		53 250	35 191	66·1%
Sex				
	Male	22 561	13 388	59·1%
	Female	30 689	21 803	71·0%
Age group, years				
	15–24	13 700	8477	61·9%
	25–34	12 636	7525	59·6%
	35–44	8724	5479	62·8%
	45–54	6984	4888	70·0%
	55–64	5733	4373	76·3%
	≥65	5473	4449	81·3%
Area of residence				
	Urban	31 481	19 483	61·9%
	Rural	21 769	15 708	72·2%
Stratum				
	1	17 987	12 296	68·4%
	2	14 185	8776	61·9%
	3	21 078	14 119	67·0%

Data are n or %.

9066 (25·8%) of 35 191 participants were screen-positive; of these, 3566 (39·3%) were positive by abnormal chest X-ray only, 3435 (37·9%) were positive by symptoms only, 1733 (19·1%) were positive by symptoms and abnormal chest X-ray, and 332 (3·7%) had no symptoms and did not have a chest X-ray (table 2). A greater proportion of men (3849 [28·7%] of 13 388) than women (5217 [23·9%] of 21 803) were screen-positive (p<0·0001). The proportion of participants who were screen-positive differed significantly between age groups and increased with increasing age from 12·6% (1064 of 8477) in people aged 15–24 years to 37·2% (1625 of 4373) in those aged 55–64 years and 47·3% (2104 of 4449) in those aged 65 years or older. More men than women were screen-positive by chest X-ray only (1699 [44·1%] of 3849 *vs* 1867 [35·8%] of 5217; p<0·0001), and more women than men were screen-positive by symptoms only (2149 [41·2%] *vs* 1286 [33·4%]; p<0·0001; table 2).

**Table 2:** Screening outcomes by sex, age group, area of residence, strata, and HIV status

		**Total screen-positive participants**	**Symptoms only**		**Abnormal chest X-ray only**		**Symptoms and abnormal chest X-ray**		**No symptoms and without chest X-ray**	
			Participants	p value	Participants	p value	Participants	p value	Participants	p value
Total		9066	3435 (37·9%)	..	3566 (39·3%)	..	1733 (19·1%)	..	332 (3·7%)	..
Sex		..	..	<0·0001	..	<0·0001	..	<0·0001	..	<0·0001
	Male	3849	1286 (33·4%)	..	1699 (44·1%)	..	818 (21·3%)	..	46 (1·2%)	..
	Female	5217	2149 (41·2%)	..	1867 (35·8%)	..	915 (17·5%)	..	286 (5·5%)	..
Age group, years		..	..	<0·0001	..	<0·0001	..	<0·0001	..	<0·0001
	15–24	1064	603 (56·7%)	..	310 (29·1%)	..	75 (7·0%)	..	76 (7·1%)	..
	25–34	1381	710 (51·4%)	..	411 (29·8%)	..	159 (11·5%)	..	101 (7·3%)	..
	35–44	1393	582 (41·8%)	..	500 (35·9%)	..	268 (19·2%)	..	43 (3·1%)	..
	45–54	1499	540 (36·0%)	..	622 (41·5%)	..	319 (21·3%)	..	18 (1·2%)	..
	55–64	1625	506 (31·1%)	..	706 (43·4%)	..	390 (24·0%)	..	23 (1·4%)	..
	≥65	2104	494 (23·5%)	..	1017 (48·3%)	..	522 (24·8%)	..	71 (3·4%)	..
Area of residence		..	..	<0·0001	..	0·31	..	<0·0001	..	<0·0001
	Urban	4214	1714 (40·7%)	..	1637 (38·8%)	..	667 (15·8%)	..	196 (4·7%)	..
	Rural	4852	1721 (35·5%)	..	1929 (39·8%)	..	1066 (22·0%)	..	136 (2·8%)	..
Strata		..	..	<0·0001	..	0·51	..	<0·0001	..	<0·0001
	1	1931	792 (41·0%)	..	773 (40·0%)	..	250 (12·9%)	..	116 (6·0%)	..
	2	1882	786 (41·8%)	..	720 (38·3%)	..	290 (15·4%)	..	86 (4·6%)	..
	3	5253	1857 (35·4%)	..	2073 (39·5%)	..	1193 (22·7%)	..	130 (2·5%)	..
HIV status*		..	..	<0·0001	..	NA	..	<0·0001	..	0·13
	HIV-positive	1647	642 (39·0%)	..	581 (35·3%)	..	375 (22·8%)	..	49 (3·0%)	..
	HIV-negative	5414	2167 (40·0%)	..	2060 (38·0%)	..	989 (18·3%)	..	198 (3·7%)	..
	Unknown	2005	626 (31·2%)	..	925 (46·1%)	..	369 (18·4%)	..	85 (4·2%)	..

Data are n or n (%) unless otherwise stated. NA=not applicable.

* HIV status determined by self-report or dried blood spot testing result; unknown in absence of both.

965 (14·8%) of 6523 chest X-rays were read as not abnormal by medical officers but as abnormal suggestive of tuberculosis by a radiologist, and 197 (20·4%) of these participants submitted sputum samples as they were symptom-screen positive. 178 (0·5%) of 35 191 participants were on tuberculosis treatment at the time of the survey and 2964 (8·4%) reported receiving tuberculosis treatment previously. HIV status was known for 26 877 (76·4%) of 35 191 participants and 4588 (17·1%) were HIV-positive. 1647 (23·3%) of 7061 participants who were screen-positive with known HIV status were HIV-positive. Data on ART were not available.

Of the 9066 participants who were screen-positive, 7509 (82·8%) had valid Xpert MTB/RIF Ultra results and 6951 (76·7%) had valid *M tuberculosis* complex culture results. 220 participant samples were culture-positive for *M tuberculosis* complex and 223 were positive on Xpert MTB/RIF Ultra assay (table 3). Of these 223 samples, seven (3·1%) were rifampicin resistant. 144 participants were positive on both Xpert MTB/RIF Ultra and culture, 66 on Xpert MTB/RIF Ultra only, and 76 on culture only. 145 (2·0%) of 7347 samples grew non-tuberculous mycobacteria on culture, and 396 (5·4%) were contaminated. All negative culture controls passed in all culture batches processed. Trace results accounted for 71 (1·0%) of 7286 Xpert MTB/RIF Ultra-negative results. Of the 71 participants with trace results, 26 (36·6%) were culture-positive and 38 (53·5%) were culture-negative (table 3).

**Table 3:** Culture and Xpert MTB/RIF Ultra results among participants who were eligible for sputum analysis

		**Culture-positive for** Mycobacterium tuberculosis	**Culture-negative for** M tuberculosis	**Culture contaminated**	**Culture grew non-tuberculous mycobacteria**	**Culture rejected or sputum not collected**	**Total**
Xpert MTB/RIF Ultra-positive		144	66	9	0	4	223
	High	33	0	0	0	1	34
	Medium	55	19	3	0	1	78
	Low	30	24	3	0	3	60
	Very low	26	22	3	0	0	51
Xpert MTB/RIF Ultra-negative		74	6460	383	145	224	7286
	Trace*	26	38	4	0	3	71
Xpert MTB/RIF Ultra invalid		0	11	0	0	1	12
Xpert MTB/RIF Ultra rejected or sputum not collected		2	49	4	0	1490	1545
Total		220	6586	396	145	1719	9066

* In this survey, Xpert MTB/RIF Ultra trace results were classified as negative.

234 (0·7%) participants were identified as having bacteriologically confirmed pulmonary tuberculosis; 220 were culture-positive for *M tuberculosis* complex and 14 were culture-negative but Xpert MTB/RIF Ultra-positive with an abnormal chest X-ray suggestive of active tuberculosis and no history of tuberculosis. Overall, 144 (65·5%) of 220 participants with culture-positive *M tuberculosis* complex were Xpert MTB/RIF Ultra-positive. 98 (41·8%) of 234 survey participants with tuberculosis reported at least one symptom, with the most frequent symptoms being cough (69 [29·5%] participants) and night sweats (51 [18·8%]). 217 (92·7%) of 234 participants had an abnormal chest X-ray. 135 (57·7%) had an abnormal chest X-ray without symptoms, 82 (35·0%) had an abnormal chest X-ray and at least one symptom, 16 (6·8%) had symptoms only, and one (0·4%) had no symptoms and had no chest X-ray. 77 (56·6%) of the 136 participants without symptoms were men aged 25–34 years, and 25 (18·6%) had previously been treated for tuberculosis. Participants who were screen-positive by abnormal chest X-ray only were eight times more likely to have bacteriologically confirmed pulmonary tuberculosis than those who were screen-positive by symptoms only (appendix p 2). 62 (26·5%) of the 234 participants with bacteriologically confirmed pulmonary tuberculosis reported previous tuberculosis, and ten (4·3%) were on tuberculosis treatment at the time of the survey. HIV status was known for 191 (81·6%) participants with bacteriologically confirmed pulmonary tuberculosis, and 55 (28·8%) were HIV-positive (median age 36 years [IQR 32–43]). More participants with tuberculosis and HIV co-infection (31 [56·4%] of 55) had symptoms than participants without HIV infection (53 [39·0%] of 136).

After statistical adjustments, our best performing model estimated the prevalence of bacteriologically confirmed pulmonary tuberculosis among people aged 15 years or older to be 852 cases (95% CI 679–1026) per 100 000 population (table 4). This estimate was at least 17% higher than the models without adjustment (appendix p 3). The estimated prevalence of bacteriologically confirmed pulmonary tuberculosis was approximately 1·6 times higher in men than in women, and was highest in people aged 35–44 years and those aged 65 years or older (table 4). Prevalence was lower in stratum 1 than in strata 2 and 3. The prevalence to case notification ratio was 1·75 overall; it was higher in men than in women and reached almost 3·00 in people aged 15–24 years and those aged 65 years or older. When extrapolated to all forms of tuberculosis and for all ages, the prevalence was estimated to be 737 cases (95% CI 580–890) per 100 000 population. Estimated prevalence of bacteriologically confirmed pulmonary tuberculosis among people with HIV was 1734 cases (95% CI 1219–2249) per 100 000 population, and among people without HIV was 900 cases (691–1108) per 100 000 population. When participants who were either culture-positive (n=220) or Xpert MTB/RIF Ultra-positive (n=223) were classed as having bacteriologically confirmed pulmonary tuberculosis, the estimated cluster-level prevalences of tuberculosis were very similar to and within the bounds of the estimate using the survey case definition (appendix p 3).

**Table 4:** Estimated prevalence of pulmonary tuberculosis in people aged 15 years or older, and prevalence to case notification ratios

		**Number of survey cases**	**Estimated prevalence, cases per 100 000 population (95% CI)**	**Tuberculosis case notification rate per 100 000 population in 2018**	**Prevalence to case notification ratio**
Total		234 (100·0%)	852 (679–1026)	486	1·75
Sex					
	Male	124 (53·0%)	1094 (835–1352)	578	1·89
	Female	110 (47·0%)	675 (494–855)	398	1·70
Age group, years					
	15–24	23 (9·8%)	432 (232–632)	149	2·91
	25–34	54 (23·1%)	902 (583–1221)	562	1·61
	35–44	48 (20·5%)	1107 (703–1511)	716	1·55
	45–54	38 (16·2%)	1063 (682–1443)	639	1·66
	55–64	30 (12·8%)	845 (505–1186)	517	1·63
	≥65	41 (17·5%)	1104 (680–1528)	383	2·88
Strata					
	1 (low prevalence)	34 (14·5%)	338 (220–457)	..	..
	2 (medium prevalence)	76 (32·4%)	933 (548–1318)	..	..
	3 (high prevalence)	124 (53·0%)	1236 (945–1526)	..	..
HIV status*					
	HIV-positive	98 (41·9%)	1734 (1219–2249)	..	..
	HIV-negative	93 (39·7%)	900 (691–1108)	..	..

Data are n (%) unless otherwise stated. Estimates are from the model using multiple imputation and inverse probability weighting.

* HIV status was unknown in 43 survey cases.

Of 98 participants identified as having bacteriologically confirmed pulmonary tuberculosis who reported at least one symptom, 41 (41·8%) had sought care before the survey. Among these, eight (19·5%) participants had already been diagnosed with tuberculosis and started on treatment. Of the 57 (58·2%) participants who had not already sought care, 38 (66·6%) were planning to seek care, eight (14·0%) regarded their symptoms as trivial, eight (14·0%) had not sought care due to distance to the clinic, travel costs, or crowded clinics, and three (5·3%) did not report a specific reason.

## Discussion

The estimated burden of bacteriologically confirmed pulmonary tuberculosis among people aged 15 years or older in South Africa in 2017–19 was 852 cases per 100 000 population. Prevalence was higher in men than in women, with a large difference in the prevalence to case notification ratio between sexes; a finding that is consistent with the literature.^16^ The prevalence to case notification ratio, a proxy of case-finding performance, indicated that the estimated number of undiagnosed or unreported tuberculosis cases was highest in people aged 15–24 years and those aged 65 years or older. Prevalence was highest in people aged 35–44 years and those aged 65 years or older. However, given the young population of South Africa, the number of cases in people aged 15–34 years represents a huge current and potentially future recurrent tuberculosis burden. Based on these survey data, post-survey incidence estimates for the main year of the survey (2018) were revised upwards by WHO to 677 cases (95% CI 472–919) per 100 000 population, albeit with considerable overlap in uncertainty intervals with the revised pre-survey estimate of 520 cases (373–692) per 100 000 population.^17^

The majority (57·7%) of participants with bacteriologically confirmed pulmonary tuberculosis had an abnormal chest X-ray and no reported symptoms, and slightly more than one-third had symptoms and an abnormal chest X-ray. The finding that more than half of all participants with pulmonary tuberculosis in this setting did not report symptoms is not new.^18^ However, it further highlights the importance of including more sensitive screening tools (ie, chest X-rays) within active case-finding activities to increase the potentially earlier detection of tuberculosis cases, given their accuracy when compared with symptoms.^19^ Exclusion of chest X-ray screening, or a sequential serial positive screening algorithm starting with symptom screening, would have missed these cases. Reserving chest X-rays for only those without symptoms would have detected all cases, without the need for the 5168 chest X-rays done in participants who reported symptoms. This finding has important cost implications when considering active case-finding screening algorithms for tuberculosis programmes. It is also particularly noteworthy when attempting to reach groups such as men who might not readily report tuberculosis symptoms or participate in interventions perceived as lengthy or time consuming. Chest X-ray uptake in this survey was high, implying that, with clear ethical and safety protocols, chest X-ray screening could be widely acceptable in systematic screening in communities.

Although this was a cross-sectional survey, the burden of bacteriologically confirmed pulmonary tuberculosis in people who did not report symptoms was not insubstantial. This potential pool of infection, which is not identified by the routine control programme, could be driving transmission of tuberculosis in communities with persistently high tuberculosis burdens,^20^ and where the observed impact of tuberculosis interventions is slow, such as in South Africa, where the rate of decline of tuberculosis incidence has decreased.^6^ In our study, people who only had an abnormal chest X-ray were eight times more likely to have tuberculosis than those with symptoms only. Although these individuals might eventually be detected and treated, the detection delay might have an impact on transmission.^20^, ^21^ This detection delay is further exacerbated by patient and health system delays when such individuals become symptomatic. For example, in this survey, less than half of symptomatic individuals with tuberculosis reported seeking care. Even after seeking care, not all patients with tuberculosis successfully navigate and appropriately exit the tuberculosis care cascade.^22^

Although HIV is a key driver of tuberculosis in this setting, we found many people with tuberculosis who were HIV-negative and therefore less likely to seek care and be detected. In addition, with wide-scale roll-out of the HIV programme and more than 4 million people living with HIV receiving ART in 2017,^5^ people living with HIV are likely to have more opportunities to access and engage with the health system and to have symptoms detected and investigated, as evidenced by the higher tuberculosis and HIV co-infection rate reported by the national tuberculosis control programme (58·0%)^6^ than in our survey (23·3%). Therefore, greater effort is required to reach those with tuberculosis who are HIV-negative in communities.

This study has some limitations. Participation and sputum collection rates were low. In future surveys, methods to improve community engagement and survey participation,^5^ sputum collection rate, and other survey indicators should be explored, to limit the potential bias linked to missing data. However, estimates with different analytical models did not considerably vary, thus reducing the likelihood of any potential bias due to missing data. HIV testing was restricted to those eligible for sputum examination, and testing rates, although low, were consistent with other surveys in South Africa that also used dried blood spot samples; 52% of the eligible population were interviewed and tested in the Demographic and Health Survey (2016),^23^, and the testing rate was only 61% in the fifth National HIV Survey (2017).^5^ Universal rapid HIV testing could have increased testing uptake, giving a more accurate estimate of co-infection, opportunity for linkage to treatment for those testing positive, and integration of the tuberculosis and HIV programmes. Future surveys, especially those conducted in high-prevalence HIV settings, should include rapid HIV testing and mechanisms for linkage to treatment, consistent with international ethical standards. Although medical officers in this study were asked to increase the sensitivity of chest X-ray reading, some potential (albeit non-fulminant asymptomatic) cases might have been missed. Future surveys should plan central chest X-ray reading for all images, to maximise quality control of the chest X-ray reading process and gain a better estimate of the potential under-reading in the field.

This survey was one of the first to use both Xpert MTB/RIF Ultra and liquid culture in parallel. Given the lower specificity of Xpert MTB/RIF Ultra, especially in the context of active case finding, we could not assume all Xpert MTB/RIF Ultra-positive results were diagnostic of active tuberculosis.^24^, ^25^ Xpert MTB/RIF Ultra might detect people who had tuberculosis previously, as well as those who had been infected but contained the infection and do not have active tuberculosis at the time of testing. Prevalence derived from only Xpert MTB/RIF Ultra would not equate to tuberculosis disease burden, but rather the prevalence of *M tuberculosis* DNA, which is likely to be an overestimation of disease burden, particularly in countries with high tuberculosis burdens. Culture, the diagnostic reference standard, also has its limits. False-negative cultures are possible in some samples with low bacterial load. Use of a low positive control in future surveys could provide information on the laboratories’ capability of culturing these samples and reduce the probability of false-negative results. However, satisfactory laboratory quality indicators in this survey suggest that false-negative results were minimal. Reasons for Xpert MTB/RIF Ultra-positive, culture-negative samples could therefore be a combination of the individual not having active disease (ie, having history of previous disease) as well as false-negative cultures in the presence of low bacterial load in the samples. We believe that by restricting the case definition to those with only an Xpert MTB/RIF Ultra-positive outcome (without a history of previous tuberculosis) identifies most people with tuberculosis who do not have culture confirmation. Although not everyone with a positive Xpert MTB/RIF Ultra result in this survey was classified as having tuberculosis, they were managed in accordance with national tuberculosis guidelines.

This study showed that tuberculosis remains an important public health issue in South Africa, due to a high tuberculosis burden and many undetected cases. Prevalence was higher in men than in women, more than half of those with tuberculosis did not report typical symptoms, and most cases were HIV-negative, possibly reflecting the great effect of a strong HIV programme to find and treat people with HIV with tuberculosis co-infection. Tuberculosis in young people (aged 15–24 years) and older adults (aged 65 years or older) was also largely undetected by the national tuberculosis control programme, indicating the need to improve engagement with and awareness in these population groups. Prioritising chest X-ray screening in case-finding strategies could potentially improve case detection. The use of molecular diagnostic tests in active case finding needs closer examination, given the possibility of providing false-positive results, especially in settings with high tuberculosis burden. Targeted interventions with more effective demand-creation strategies are recommended to increase health-care seeking, especially among people with symptoms.

**This online publication has been corrected. The corrected version first appeared at thelancet.com/infection on May 27, 2022**

## Data sharing

Individual, deidentified participant data, including data dictionaries, may be shared. Templates of the informed consent forms may be shared upon request. The data will be available following publication, with no end date, and will be shared with anyone who wishes to access them with a clear data sharing agreement, for any purpose of analyses. For data access, please contact the corresponding author and the tuberculosis programme at the National Department of Health in South Africa.

## Declaration of interests

We declare no competing interests.

## References

[1] South African National AIDS Council (2017). National strategic plan for HIV, TB and STIs 2017–2022. https://nsp.sanac.org.za.

[2] National Health Laboratory Service TB GeneXpert. https://www.nhls.ac.za/priority-programmes/tb-genexpert/.

[3] South African National AIDS Council The national wellness campaign: Cheka Impilo. https://sanac.org.za/the-national-wellness-campaign-cheka-impilo/.

[4] South African Department of Health (2019). DOH/PEPFAR best practices meeting: HIV patient linkage and return to care. https://za.usembassy.gov/wp-content/uploads/sites/19/HIV-Patient-Linkage-and-Return-Back-to-Care_Zukiswa-Pinini_National-Department-of-Health-1.pdf.

[5] Simbayi LC, Zuma K, Zungu N (2019).

[6] WHO (2018).

[7] WHO (2011).

[8] WHO (2014).

[9] South African Department of Health (2014). National tuberculosis management guidelines, South Africa. http://www.kznhealth.gov.za/family/NTCP_Adult_TB_Guidelines_2014.pdf.

[10] Dorman SE, Schumacher SG, Alland D (2018). Xpert MTB/RIF Ultra for detection of *Mycobacterium tuberculosis* and rifampicin resistance: a prospective multicentre diagnostic accuracy study. Lancet Infect Dis.

[11] Harris PA, Taylor R, Minor BL (2019). The REDCap consortium: building an international community of software platform partners. J Biomed Inform.

[12] Harris PA, Taylor R, Thielke R, Payne J, Gonzalez N, Conde JG (2009). Research electronic data capture (REDCap)—a metadata-driven methodology and workflow process for providing translational research informatics support. J Biomed Inform.

[13] Floyd S, Sismanidis C, Yamada N (2013). Analysis of tuberculosis prevalence surveys: new guidance on best-practice methods. Emerg Themes Epidemiol.

[14] UN Department of Economic and Social Affairs (2015). The world population prospects: 2015 revision. https://www.un.org/en/development/desa/publications/world-population-prospects-2015-revision.html.

[15] Borgdorff MW (2004). New measurable indicator for tuberculosis case detection. Emerg Infect Dis.

[16] Horton KC, MacPherson P, Houben RM, White RG, Corbett EL (2016). Sex differences in tuberculosis burden and notifications in low- and middle-income countries: a systematic review and meta-analysis. PLoS Med.

[17] WHO (2020).

[18] WHO (2021).

[19] WHO (2021).

[20] Frascella B, Richards AS, Sossen B (2021). Subclinical tuberculosis disease—a review and analysis of prevalence surveys to inform definitions, burden, associations, and screening methodology. Clin Infect Dis.

[21] Drain PK, Bajema KL, Dowdy D (2018). Incipient and subclinical tuberculosis: a clinical review of early stages and progression of infection. Clin Microbiol Rev.

[22] Naidoo P, Theron G, Rangaka MX (2017). The South African tuberculosis care cascade: estimated losses and methodological challenges. J Infect Dis.

[23] South African Department of Health (2019). South Africa Demographic and Health Survey 2016. https://dhsprogram.com/pubs/pdf/FR337/FR337.pdf.

[24] Mishra H, Reeve BWP, Palmer Z (2020). Xpert MTB/RIF Ultra and Xpert MTB/RIF for diagnosis of tuberculosis in an HIV-endemic setting with a high burden of previous tuberculosis: a two-cohort diagnostic accuracy study. Lancet Respir Med.

[25] Zifodya JS, Kreniske JS, Schiller I (2021). Xpert Ultra versus Xpert MTB/RIF for pulmonary tuberculosis and rifampicin resistance in adults with presumptive pulmonary tuberculosis. Cochrane Database Syst Rev.

