# Correction to *Lancet Infect Dis* 2022; published online May 17. https://doi.org/10.1016/S1473-3099(22)00149-9

**DOI:** 10.1016/S1473-3099(22)00350-4

**Published:** 2022-07

*Moyo S, Ismail F, Van der Walt M, et al. Prevalence of bacteriologically confirmed pulmonary tuberculosis in South Africa, 2017–19: a multistage, cluster-based, cross-sectional survey.* Lancet Infect Dis *2022; published online May 17. https://doi.org/10.1016/S1473-3099(22)00149-9*—In this Article, the spelling of author Olanrewaju Oladimeji's name was incorrect. This correction has been made to the online version as of May 27, 2022, and will be made to the printed version.

