# Supplementary material for: Prevalence of bacteriologically confirmed pulmonary tuberculosis in South Africa, 2017–19: a multistage, cluster-based, cross-sectional survey
Source: Lancet Infect Dis. 2022 Aug;22(8):1172–80. doi: 10.1016/S1473-3099(22)00149-9 (PMC9300471; doi:10.1016/S1473-3099(22)00149-9)
Supplement: Supplementary appendix [file mmc1.pdf]

THE LANCET
Infectious Diseases

Supplementary appendix

This appendix formed part of the original submission and has been peer reviewed. We post it as supplied by the authors.

Supplement to: Moyo S, Ismail F, Van der Walt M, et al. Prevalence of bacteriologically confirmed pulmonary tuberculosis in South Africa, 2017–19: a multistage, cluster-based, cross-sectional survey. *Lancet Infect Dis* 2022; published online May 17. https://doi.org/10.1016/S1473-3099(22)00149-9.

<u>Supplementary Material</u>

Web Appendix:

Contents

Contents

Analytical methods 1

Web Appendix Table 1: Case disaggregated by screening category and HIV status 2

Web Appendix Table 2: Estimated bacteriologically confirmed pulmonary TB prevalence per 100000 population by analytical model 3

Web Appendix Table 3: Estimated prevalence of TB per 100000 population (cluster-level) in ≥ 15 years, by diagnostic method 3

Web Appendix references 4

Survey questionnaires : South African National TB Prevalence Survey- 2016-2017 5

Analytical methods

Analytical methods follow international recommendations on best analytical practices issued by the WHO Global Task Force on TB Impact Measurement,[1] and have also been used in the analysis of numerous national TB prevalence surveys already.[2-6] The two main points that require attention in the analysis of these population based TB prevalence surveys are to properly account for the clustered sampling design (and its associated design effect) and to investigate the potential bias in the produced estimates due to the missingness of data at the various stages of the survey. The sampling design of the survey is such that it creates a self-weighted survey sample drawn from the general population, since clusters have been selected to be proportional to size and the sample size of each cluster is similar. This design simplifies the analysis that does not need to account for sampling weights.

We started the explorative analysis by fitting a cluster-level regression model, which accounts for the sampling design but does not allow for missing value imputation and any investigations for the association of being a survey case of TB and any possible risk factors. For this model we aggregate individual-level data to the level of the cluster, so that the cluster becomes the unit of analysis. TB prevalence among survey participants is calculated separately for each cluster (e.g., 3 cases out of 450 participants), and the average cluster-level prevalence is then calculated.

We then fit three individual-level regression models as part of the explorative analyses, to understand better how patterns of data missingness at different stages of the survey might have introduced biases to the model estimates. The figure below presents how each model accounts for the missingness of survey data generated at each part of the survey, from the full enumerated population to the eligible population and then finally to the population that actually participated in the survey split into those individual eligible for sputum examination (i.e. screened positive by either a chest X-ray or a symptoms interview) and those not eligible for sputum examination.

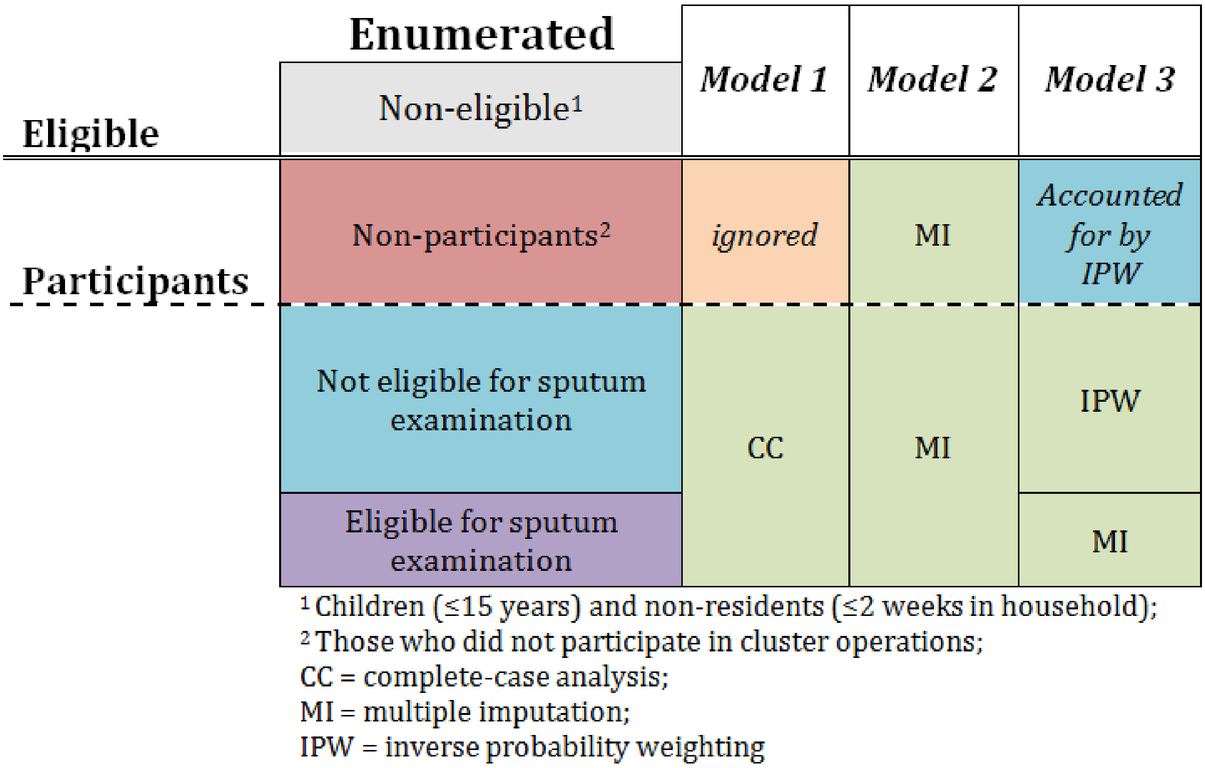

Logistic regression models with robust standard errors are used for all three models, accounting for the clustering by correcting standard errors, confidence intervals and p-values. Model 1 assumes the pattern of missingness is missing completely at random (MCAR) and restricts the analysis to participants with complete case data. MCAR

is too strong an assumption to make and results from this model are used only as part of the exploration of observed data. Model 2 includes all eligible individuals irrespective of whether they participated in cluster operations or not. Multiple imputation is used for all missing values of relevant variables and the pattern of missingness is assumed to be missing at random (MAR). The model essentially uses observed patterns of data within each stratum (defined by all possible combinations of individual characteristics that are included in the imputation model), to predict values for individuals for whom data are missing (either in the primary outcome or the risk factors of the imputation model). Model 2 is the one that imputes the most data and is again mostly used as part of the exploration of the observed survey data. Finally, Model 3 uses a combination of multiple imputation among a small group of individuals (those eligible for sputum examination), and inverse probability weighting for participants not eligible for sputum examination and non-participants. The results of Model 3 are recommended as those that are most reliable and the ones to be used as final results from the survey.

For the results reported in the paper, Model 3 (MI+IPW) adhered to the following:

- A bacteriologically confirmed TB case was defined as an eligible screen-positive participant with *M.tb* complex culture positive samples, and when culture was not positive i.e., negative, contaminated or not done, cases were Xpert Ultra positive samples with an abnormal CXR (as determined by a central panel of three readers) and no history of current or prior TB.

- The primary outcome for each survey participant is whether they are a bacteriologically confirmed TB case (yes/no). If this was missing, then the model would impute values for either the primary outcome or the risk factors of the imputation model (using the chained equations approach). The imputation model was defined using these variables: stratum, age group, cough of more than 2 weeks, past history of TB, HIV status, race and sex. The imputation model was built based on associations of the risk factors either with the primary outcome or the outcome of missingness.

- Multiple imputation results used and reported are based on 25 imputed datasets (more imputed datasets were not necessary as these produced very similar results to the 25), after a burn-in step of the first 20 imputed datasets, using the suite of *mi* commands in Stata (v16).

- For the IPW analysis, probability weights were generated by dividing the number of total eligible individuals by the total number of participants for each combination of cluster (1-110), sex (male, female) and age group (15-24, 25-34, 35-44, 45-54, 55-64, ≥65 years). In total, 1320 different weights were then applied to all survey participants who were screened to correct for differentials in participation by cluster, sex and age. Higher weights are assigned to lower participation (or observed data).

- "Participation rate" is defined as the probability of those who consented to participate divided by those eligible to participate in the survey (i.e., ≥15 years and resident within the households of the selected clusters). In this survey, the participation rate was 66.1% (i.e., 35,191 participated out of 53,250 eligible population). Therefore, the non-participation rate was 33.9%.

Web Appendix Table 1: Case disaggregated by screening category and HIV status

			TB survey case		
**Screening category**	**Screen positive participants**	**Survey cases (%)**	**HIV-positive***	**HIV-negative***	**HIV-unknown***
Symptoms only	3435	16 (0.5%)	5	9	2
Abnormal CXR only	3566	135 (3.8%)	23	83	29
Symptoms and abnormal CXR	1733	82 (4.7%)	26	44	12
No symptoms, No CXR done	332	1 (0.3%)	1	0	0
Total	9066	234	55	136	43

CXR: chest X-ray.
*HIV status determined by self-report or by (DBS) dried blood spot test result, unknown in absence of both

Web Appendix Table 2: Estimated bacteriologically confirmed pulmonary TB prevalence per 100000 population by analytical model

Model	Prevalence per 100000 population (95% CI)
Cluster-level	707 (617-798)
Model 1: Robust standard errors without multiple imputation	707 (552-863)
Model 2: Robust standard errors with multiple imputation	733 (579-887)
Model 3: Robust standard errors with multiple imputation and inverse probability weighting	852 (679-1026)

Web Appendix Table 3: Estimated prevalence of TB per 100000 population (cluster-level) in ≥ 15 years, by diagnostic method

	Culture-positive only		Xpert Ultra positive only		Survey case definition	
	Number of cases	Prevalence per 100000 population (95% CI)	Number of cases	Prevalence per 100000 population (95% CI)	Number of cases	Prevalence per 100000 population (95% CI)
**Total**	220	665 (557-753)	223	663 (576-750)	234	707 (617-798)
**Sex**						
Male	116	919 (751-1086)	123	958 (789-1127)	124	982 (809-1155)
Female	104	509 (411-606)	100	481 (387-575)	110	538 (437-638)
**Age group (years)**						
15-24	22	270 (157-383)	19	232 (128-337)	23	282 (167-398)
25-34	51	709 (514-904)	48	661 (474-848)	54	750 (550-951)
35-44	43	831 (583-1079)	51	971 (704-1237)	48	927 (665-1189)
45-54	35	766 (512-1020)	51	1091 (792-1390)	38	832 (567-1096)
55-64	29	717 (456-978)	29	673 (424-922)	30	742 (476-1007)
≥ 65	40	1010 (697-1323)	26	634 (390-878)	41	1035 (718-1352)

Web Appendix references

1. Floyd, S., Sismanidis, C., Yamada, N. *et al.* Analysis of tuberculosis prevalence surveys: new guidance on best-practice methods. *Emerg Themes Epidemiol* 10**,** 10 (2013).

2. Bonsu F, Addo KK, Alebachew Z, Gyapong J, Badu-Peprah A, Gockah R, Hanson-Nortey NN, Law I, Tadolini M, Onozaki I, Sismanidis C, Owusu-Dabo E. National population-based tuberculosis prevalence survey in Ghana, 2013. Int J Tuberc Lung Dis. 2020 Mar 1;24(3):321-328.

3. Nguyen HV, Tiemersma EW, Nguyen HB, Cobelens FGJ, Finlay A, Glaziou P, Dao CH, Mirtskhulava V, Nguyen HV, Pham HTT, Khieu NTT, de Haas P, Do NH, Nguyen PD, Cung CV, Nguyen NV. The second national tuberculosis prevalence survey in Vietnam. PLoS One. 2020 Apr 23;15(4):e0232142. Erratum in: PLoS One. 2020 Jul 16;15(7):e0236532.

4. Lansang MAD, Alejandria MM, Law I, Juban NR, Amarillo MLE, Sison OT, Cruz JRB, Ang CF, Buensalido JAL, Cañal JPA, Castillo-Carandang NT, Cordero CP, Gaviola DMG, Ladia MAJ, Mantaring JBV 3rd, Mendoza MT, Salamat MSS, Lam HY, Tadolini M, Garfin AMCG; Philippine NTPS 2016 Group. High TB burden and low notification rates in the Philippines: The 2016 national TB prevalence survey. PLoS One. 2021 Jun 4;16(6):e0252240.

5. Migambi P, Gasana M, Uwizeye CB, Kamanzi E, Ndahindwa V, Kalisvaart N, Klinkenberg E. Prevalence of tuberculosis in Rwanda: Results of the first nationwide survey in 2012 yielded important lessons for TB control. PLoS One. 2020 Apr 23;15(4):e0231372.

6. Enos M, Sitienei J, Ong'ang'o J, Mungai B, Kamene M, Wambugu J, Kipruto H, Manduku V, Mburu J, Nyaboke D, Ngari F, Omesa E, Omale N, Mwirigi N, Okallo G, Njoroge J, Githiomi M, Mwangi M, Kirathe D, Kiplimo R, Ndombi A, Odeny L, Mailu E, Kandie T, Maina M, Kasera K, Mulama B, Mugi B, Weyenga H. Kenya tuberculosis prevalence survey 2016: Challenges and opportunities of ending TB in Kenya. PLoS One. 2018 Dec 26;13(12):e0209098. Erratum in: PLoS One. 2019 Jan 25;14(1):e0211593. PMID: 30586448; PMCID: PMC6306266.

Survey questionnaires : South African National TB Prevalence Survey- 2016-2017

HOUSEHOLD QUESTIONNAIRE AND CENSUS FORM

GEOGRAPHIC PARTICULARS										
Province name										
Province number										
Small Area layer number (SAL number)										
**Household number**										
Address										

Q01_INC- HHC	**Interviewer's code**	**Enter the your fieldworker number here**

**Q01_1IHHC-CON**	**Has consent been taken?**	**Yes**	**No**

The interview can proceed only when consent for the household has been taken and the consent form(s) duly signed.

INTERVIEW DETAILS									
	**Year**		**Month**		**Day**		**Time code**	**Response code**
First visit								
Second visit								
Third visit								
Final response code								

**Time code**	**Response code**	
1 = Morning till 12:00<br>2 = 12:00-16:00<br>3 = 16:00-18:00<br>4 = 18:00-20:00<br>5 = 20:00 and later	1 = Interview completed<br>2 = Not a valid visiting point<br>3 = No one living here (unoccupied)<br>4 = Refusal by household head<br>5 = Refusal by other resident	6 = Partly completed / appointment made<br>7 = No one at home<br>8 = No one eligible to complete questionnaire<br>9 = Incapacitated<br>10 = Other

REFUSAL PARTICULARS		
NO.	QUESTIONS AND FILTERS	CODING CATEGORIES
02	At what point did the respondents refuse to take part in the survey?	AT THE GATE OR DOOR.......... 1<br>AFTER EXPLANATION OF THE SURVEY..............2<br>AFTER IDENTIFYING THE RESPONDENT.......... 3<br>DURING THE HOUSEHOLD INTERVIEW..............4<br>OTHER (SPECIFY).......................9

REFUSAL PARTICULARS		
NO.	QUESTIONS AND FILTERS	CODING CATEGORIES
03	What was the reason for the refusal?	TOO BUSY TO GRANT INTERVIEW .... 1<br>NOT AVAILABLE NOW.. 2<br>TOO LATE IN THE EVENING..3<br>DON'T PARTICIPATE IN SURVEYS ....4<br>OBJECT TO TOPIC OF THE SURVEY ....5<br>OBJECT TO PROVIDE INFORMATION OF HOUSEHOLD MEMBERS ....6<br>DO NOT ALLOW STRANGERS ON PROPERTY.......7<br>ENUMERATED IN THE RECENT POPULATION CENSUS…………………………………………......................8<br>OTHER (SPECIFY) 9

Q04_HH head	Name of the head of household	

Q05_NOPHH	**How many people live in the household?** (Include only those people who have slept in the house for at least 10 nights of the prior two weeks and normally live and share meals in the household)	

QO6_HMHHC Details of household members							
**Person number**	**Name of HH member**	**Age of HH member in years** SELECT 00 IF UNDER 1 YEAR)	**Sex** **Male=1** **Female =2**	**HH visit study number (barcode)**	**Individual census ID (from barcode)**	**Eligibility**** **1 = eligible** **2 = ineligible**	**Invitation to Survey Field Site** **1 = Accepted** **2 = Declined**
1 **(Household head)**							
2							
3							
4							
5							
6							
7							
8							
9							
10							
11							
12							

(Use extra page if needed)
** Those aged 15 years or older are eligible to participate
**Those that have slept in the household for at least 10 nights of the prior two weeks are eligible to participate

Q07 ASHHC AVAILABILITY OF SERVICES			
	QUESTIONS AND FILTERS	CODING CATEGORIES	
Q07_1	What is the main source of drinking water for members of your household?	**(SELECT 1)**<br>PIPED WATER (TAP) IN DWELLING.......... 1<br>PIPED WATER (TAP) IN SITE/YARD..........2<br>BOTTLED WATER .... 3<br>WATER CARRIER/TANKER.................... 4<br>RAIN-WATER TANK .... 5<br>BOREHOLE / WELL / SPRING................. 6<br>DAM/RIVER/STREAM........................... 7<br>PUBLIC / COMMUNAL TAP .... 8<br>OTHER ________________________9	
Q07_2	What is this household's main source of energy for cooking purposes?	**(SELECT 1)**<br>ELECTRICITY..........1<br>COAL..........2<br>WOOD..........3<br>GAS..........4<br>PARAFFIN..........5<br>ANIMAL DUNG..........6<br>OTHER..........9	
Q07_3	Does your household have any of the following (in a working condition)? (READ OPTIONS)<br>Electricity?<br>A radio?<br>A television?<br>A landline telephone<br>A cellphone?<br>A refrigerator?<br>A personal computer (PC)?<br>A washing machine?	**(SELECT THE RELEVANT OPTION)**<br>YES NO<br>ELECTRICITY.......... 1 2<br>RADIO.......... 1 2<br>TELEVISION.......... 1 2<br>TELEPHONE (landline).......... 1 2<br>CELLPHONE.......... 1 2<br>REFRIGERATOR.......... 1 2<br>PERSONAL COMPUTER.......... 1 2<br>WASHING MACHINE.......... 1 2	
Q07_4	How many rooms does your dwelling consist of?<br><br>NOTE: EXCLUDE BATHROOMS AND TOILETS.	**(SELECT VALUE)**<br>ROOMS..........[ ][ ]	
Q07_5	How many rooms in your dwelling are used for sleeping?<br><br>NOTE: A ROOM MAY ALSO HAVE ANOTHER PURPOSE BESIDES AS A BEDROOM.	**(SELECT VALUE)**<br>ROOMS FOR SLEEPING..........[ ][ ]	

Q08 IV HHCINDICATORS OF VULNERABILITY		
Q08_1	**SELECT ONLY ONE OPTION**<br>The cost of living is a concern for many families. Can you tell me what this household can afford? Which option best describes your household situation?	NOT ENOUGH MONEY FOR BASIC THINGS LIKE FOOD AND CLOTHES................................. 1<br>MONEY FOR FOOD AND CLOTHES, BUT SHORT ON MANY OTHER THINGS.................................2<br>WE HAVE MOST OF THE IMPORTANT THINGS, BUT FEW LUXURY GOODS..................................3<br>MONEY FOR EXTRA THINGS SUCH AS HOLIDAYS AND LUXURY GOODS...................................4
Q08_2	Can you please tell me what the approximate monthly income is for this household?	**(ENTER THE AMOUNT REPORTED)**<br>1. R ....<br>2. not known/ did not answer
Q08_3	**SELECT ONLY ONE OPTION**<br>In the past 4 weeks, was there ever no food to eat (of any kind) in your household, because of a lack of resources to get food??	YES.. 1<br>NO.. 2<br>DON'T KNOW .... 3<br>**(If "No" or 'Don't know" conclude the interview)**
Q08_4	**SELECT ONLY ONE OPTION**<br>How often did this happen in the past 4 weeks?	RARELY (1-2 TIMES) .... 1<br>SOMETIMES (3-10 TIMES)…………. .... 2<br>OFTEN (MORE THAN 10 TIMES)………... .. 3
Q08_5	**SELECT ONLY ONE OPTION**<br>In the past 4 weeks, did you or any household member go to sleep at night hungry because there was not enough food?	YES.. 1<br>NO.. 2<br>DON'T KNOW .... 3<br>**(If "No" or 'Don't know" –conclude the interview)**
Q08_6	**SELECT ONLY ONE OPTION**<br>How often did this happen in the past 4 weeks?	RARELY (1-2 TIMES) .... 1<br>SOMETIMES (3-10 TIMES)…………. .... 2<br>OFTEN (MORE THAN 10 TIMES)………... .. 3

INSTRUCTION: BEFORE LEAVING THE HOUSEHOLD ENSURE THAT

- **ALL ELIGIBLE INDIVIDUALS WHO HAVE AGREED TO PARTICIPATE HAVE A SURVEY FIELD SITE APPOINTMENT CARD**
- **ALL ELIGIBLE INDIVIDUALS WHO HAVE AGREED TO PARTICIPATE ARE AWARE OF THE TIME AT WHICH THEY SHOULD PRESENT AT THE SITE**

South African National TB Prevalence Survey- 2017-2019

Individual Screening interview

Q01_INC	**Interviewer's code**	**Enter the your fieldworker number here**

**Q01_INC**	**Has consent been taken?**	**Yes**	**No**

The interview can proceed only when consent has been taken and the consent form(s) duly signed.

Q02_DAT	**Date today**								
		D	D	M	M	Y	Y	Y	Y

Q03_IND	**Individual study ID**	**Scan barcode**

Q04_SEX	**Sex**	M	F
		1	2

Q05_DOB_1	**Date of Birth** (01/01/1800 if unknown)								
		D	D	M	M	Y	Y	Y	Y

Q06_DOB_2	What was your age in years at your last birthday**?**			years
	Don't know/Don't remember	999		years

Q07_RACE	Can you please describe your race? Select only one option	
	Black	1
	Coloured	2
	Indian/Asian	3
	White	4
	Other	5

Q8_COB	What is your country of birth?
	(Drop down menu with SADC countries and few other Africa countries)

Q9_MOY	What has been your **main** occupation during the past year?	
	Employed full-time(formal or informal)	1
	Employed part-time(formal or informal)	2
	Occasional/seasonal (short-term) employment	3

	Unemployed	4
	Unable to work (sick/disabled)	5
	Student	6
	Homemaker	7
	Retired	8
	Other (Specify_________)	9

If unemployed, unable to work, student, homemaker, or retired go to Q11

Q10_EOC	If employed (part time, occasional, seasonal or full-time), in what sector was this in?	Yes
	Mining	1
	Health Care	2
	Correctional services	3
	Agriculture	4
	Other (Specify_________)	5

I will now ask you some questions about your education

Q11_HEA	What is the highest level of education you have attained?	
	No Formal Education	0
	Grade 1-12(Indicate actual grade)Note Grade 8-12 is also Form 1 –form 5	1
	College/Technikon	2
	University	3

I would like to ask you some questions about smoking, alcohol, diabetes, silicosis and HIV

Q12_STP	Do you currently smoke tobacco products?	No	Yes
		0	1

If no go to Q13

Q12_1STP	How often do you smoke these products?	
	Daily	1
	Less than daily	2
	Don't know/unknown	3

Q12_2STP	On overage how much do you smoke per week?		
	Number of manufactured cigarettes		per week
	Number of hand rolled cigarettes		per week
	Number of tobacco pipes		per week
	unknown	999	per week

Q13_ALC	How often do you drink alcoholic beverages?	
	Never	0
	Once a month or less	1
	2- 4 times a month	2
	2-3 times a week	3
	4 or more times a week	4

If never go to Q15

Q13_1_ALC	How many drinks containing alcohol do you have on a typical day when you are drinking?	
	1-2 drinks	1
	3-4 drinks	2
	5-6drinks	3
	7-8 drinks	4
	10 or more	5

** 1 drink= is approximately *1 can of ordinary beer/1 glass of wine/ Carton of ordinary commercial sorghum beer*

Q15_DIA	Have you been diagnosed with diabetes?	No	Yes	Don't know
		0	1	999

If no or don't know, go to Q16

Q15_1_DIA	When were you diagnosed with diabetes?	Y	Y	Y	Y
	Don't know/Don't remember	1	8	0	0

Q15_2_DIA	Are you taking any medication(s) to treat diabetes?	No	Yes
		0	1

Q16_SIL	Have you been diagnosed with silicosis (dust disease)?	No	Yes	Don't know
		0	1	999

If no or don't know, go to Q17

Q16_1SIL	When were you diagnosed with silicosis?	Y	Y	Y	Y
	Don't know/ Don't remember	1	8	0	0

I would like to ask you some questions about HIV

Q17_HIV	Have you tested for HIV before?	No	Yes	Don't know
		0	1	999

If no go to Q18_

Q17_1HIV	When was the last time you tested?	

	In the last year	1
	1-2 years ago	2
	More than 2 years ago	3
	Don't remember	4

Q17_3HIV	What was the result?	
	Negative	0
	Positive	1
	Not willing to disclose test result	2
	Don't know	3

If Negative or not willing to disclose test result go to Q18_

Q17_4HIV	Are you on ART?	No	Yes
		0	1

I will now ask you some questions about TB

Current TB treatment

Q18_CTB	Do you know/have you ever heard about TB?	No	Yes
		0	1

If no go to Q26

Q18_1CTB	Are you currently on TB treatment? Probe and be sure the participant only refers to conventional treatment for TB	No	Yes
		0	1

If no go to Q21

Q18_2CTB	For how long have you been on treatment for TB?		
			months
		99	Don't know

Q19_RTF	Where are you currently receiving your TB treatment from?	
	Government/Community clinic	1
	Government Provincial/District hospital	2
	Private Clinic/hospital	3
	Private Pharmacy	4
	Private Doctor /GP	5

Q20_FPS	Where did you first present your symptoms?	
	Government/Community clinic	1
	Private Clinic/hospital	2
	Government Provincial/District hospital	3
	Private Pharmacy	4
	Private Doctor	5

	Traditional Healer	6
	Other (specify)	7

Previous TB treatment

Q21_TTB	Have you ever been on TB treatment before? (excludes current episode for those who are currently on treatment for TB)	No	Yes	Don't know
		0	1	999

If no go to Q25

Q22_HMT	How many times have you been on TB treatment before?(excludes current episode for those who are currently on treatment for TB)	
	Once	1
	Twice	2
	Three times	3
	More than three times	4
	Don't know	5

Q23_HLT	In your last TB episode for how many months did you take treatment? (excludes current episode for those who are currently on treatment for TB)	months	Don't know/can't remember
			999

Q24_TTB	What type of TB were you treated for?	
	Normal TB (drug sensitive TB)	1
	MDR or XDR TB	2
	Don't Know	999

Q25_IPT	Have you ever been on preventive therapy for TB (IPT)?	No	Yes	Don't Know/ can't remember
		0	1	999

I will now ask you about your current state of health

Q26_CHC	Do you currently have a cough?	No	Yes
		0	1

If no go to Q27_

Q26_2WBC	For how long have you been coughing?	
	<1 week	1
	1-2 weeks	2

	≥2 weeks	3
	Don't know/ can't remember	999

Q27_CHF	Do you currently have a fever?	No	Yes	Don't Know
		0	1	999

If no go to Q28_

Q27_1CHF	How long have you had this fever?	<2weeks	≥2weeks	Don't Know
		0	1	999

Q28_DNS	Do you currently have drenching night sweats?<br>(So that you have to change bedding or night clothes?	No	Yes
		0	1

If no go to Q30

Q29_LWU	In the last month have you lost weight unintentionally?	No	Yes	Don't Know
		0	1	999

If no to Q26, Q27, Q28, Q29, go to Q36

30_CAS	Did you consult anybody for any of these symptoms	No	Yes	N/A
		0	1	3
a	cough			
b	fever			
c	Night sweats			
d	Unintentional weight loss			

If no for all cough, fever, night sweats, unintentional weight loss, go to Q35

Q31_SHF	Where did you go for help first?	
	Government/Community clinic	1
	Private Clinic/hospital	2
	Government Provincial/District hospital	3
	Private Pharmacy	4
	Private Doctor	5
	Traditional Healer	6
	other (specify)	7

Q32_GCP	If private clinic or hospital or doctor/ pharmacy /tradition healer, did you ever go to a government/community/sputum collection point	No	Yes
		0	1

Q33_ASS	What has been done for you when you sought help?	No	Yes	N/A	Don't know

	**(Read out the response options. Multiple responses are possible.)**				
a	1 sputum sample was collected				
b	2 sputum samples were collected				
c	A CXR done				
d	Blood tests done				

If no or don't know to all options above go to Q37

Q34_1ASS	What were the results of the following tests	Negative for TB	Positive For TB	Don't know/ Did not get results	N/A
		0	1	2	3
a	sputum sample collected				
b	CXR				

Q35_RX	If results were positive for TB above, where you started on TB treatment?		Yes	No	Don't know
			1	2	3

Q36-RDNSC	What are the reasons for not seeking care for any of the symptoms? **(multiple responses possible)**	
	distance- the health centre is far from where I live	1
	Money - I had no money for transport to the health centre	2
	Relevance- I dd not consider it to be important	3
	Still planning to seek care	4
	Other specify	5

Attitude toward people with TB

Q37_KAP	How do you regard or treat people who have TB?	
	Just like everybody else	1
	I support them where I can	2
	I generally try to avoid them for fear of getting TB	3

Q38_OLH	Has a **doctor** or **nurse** or **health worker** at a **clinic** or **hospital told you** that you have or have had any of the following conditions:	No	Yes	Don't know
a	Chronic Bronchitis/ Emphysema/COPD?	0	1	99
b	Asthma?			
c	Have you had an attack of asthma in the last 12 months			
d	Compared to other people your age, do you feel you have less breath when exerting (exercising or moving a lot) yourself?			

THANK YOU FOR YOUR HELP

Important Note:

Please review the answers to the questions listed in the table below.

	Yes	No
26_1CHC Do you currently have a cough?		
Q27_1CHF Do you currently have fever of more than 2 weeks?		
Q28_DNS Do you currently have drenching night sweats?		
Q29_LWU -In the last month have you lost weight unintentionally?		

If the answer to any of the questions is "Yes", the participant must attend the Sputum Specimen station in addition to the CXR station.

Final Response code

1 = Interview completed

2 = Partially completed

3 = Other specify

		Date								Signature
		d	d	m	m	y	y	y	y	
Nurse leader										
Field Data manager										
